# A 6-month prognostic nomogram incorporating hemoglobin level for intracerebral hemorrhage in younger adults

**DOI:** 10.1186/s12883-022-03039-9

**Published:** 2023-01-06

**Authors:** Yuyan Yang, Shanshan Huang, Yuchao Jia, Guini Song, Xiaodong Ye, Kai Lu, Guo Li, Furong Wang, Suiqiang Zhu

**Affiliations:** Department of Neurology, Tongji Hospital, Tongji Medical College, Huazhong University of Science and Technology, No 1095 Jiefang Avenue, Qiaok’ou District, Wuhan, 430030 Hubei China

**Keywords:** Nomogram, Haemoglobin, Intracerebral hemorrhage, Prognosis

## Abstract

**Objective:**

Intracerebral hemorrhage (ICH) is the second most common subtype of stroke, with high mortality and morbidity. At present, there are no effective 6-month prognostic markers, particularly for younger patients. The aim of this research was to construct a new valuable prognostic nomogram model incorporating haemoglobin levels for adult patients with ICH.

**Methods:**

Patients aged between 18 and 50 presenting with intracerebral haemorrhage at the Tongji Hospital, Tongji Medical College of Huazhong University of Science and Technology between January 1st 2012 and December 31st 2018 were included in this retrospective study. Independent factors of prognosis were identified by univariate and multivariate logistic regression analyses, and a new nomogram model was constructed and validated. The clinical value of the nomogram model was subsequently explored utilizing decision curve analysis and clinical impact curves.

**Results:**

In total, 565 patients were enrolled in this study, 117 (20.7%) of whom developed an unfavourable prognosis. Infratentorial lesion (adjusted odds ratio [aOR] = 3.708, 95% confidence interval [CI], 1.490–9.227; *P* = 0.005) was the most significant unfavourable outcome. Age ([aOR] = 1.054; 95% CI, 1.014–1.096; *P* = 0.008), hematoma volume (aOR = 1.014, 95% CI, 1.002–1.027; *P* = 0.024), haemoglobin (aOR = 0.981, 95% CI, 0.969–0.993; *P* = 0.002), blood glucose (aOR = 1.135, 95% CI, 1.037–1.241; *P* = 0.005) and NIHSS (aOR = 1.105, 95% CI, 1.069–1.141; *P* < 0.001) were independent risk factors. Based on these 6 factors, the nomogram can be employed to predict early functional prognosis with high accuracy (AUC 0.791). Decision curve analysis and clinical impact curves showed an increased net benefit for utilizing the nomogram.

**Conclusion:**

The haemoglobin level at admission may be an easily overlooked factor in clinical work. This new nomogram model could be a promising and convenient tool to predict the early functional prognosis of adults with ICH. More prospective multicentre studies are needed to validate these findings.

## Introduction

Intracerebral hemorrhage (ICH) is a devastating stroke subtype, accounting for 10–27% of all strokes [1]. Adults are the main population of intracerebral hemorrhage, with 40–70 years of age as the main age of onset,and it increases with age [2]. Due to the high mortality rate and disability rate of ICH [3], more attention should be given to the functional outcome of younger adults to avoid a consequent increase in the socioeconomic burden. A young adult patient with ICH is usually defined as aged between 18 and 50 years, although the pre cise age range differs between studies and experts [3]. Several studies have reported that only 34.9 − 39.9% of younger patients reach a favourable short-term outcome, and 39.0–59.5% of patients attain a favourable long-term outcome [4–8].

Many studies conducted in younger adults have explored common risk factors related to functional outcomes, such as age, increasing initial National Institutes of Health Stroke Scale score and ventricular extension of the hemorrhage [9–11]. Meanwhile, a meta-analysis found that haemoglobin is closely associated with mortality in patients with intracerebral hemorrhage, suggesting that it may be a neglected blood indicator [12]. However, none of the studies integrated both common risk factors and haemoglobin-related data into a systematic assessment methodology applied to younger adults. Therefore, such a systematic prognostic model applied to younger adults is needed for risk stratification guidelines for treatment and rehabilitation.

At present, nomograms are a useful statistical tool for assessing and calculating the precise risk of individual patients for both short-term and long-term outcomes [13]. However, there is no nomogram for younger adults with intracerebral hemorrhage. To better distinguish important and overlooked predictors of unfavourable outcomes, we conducted a retrospective study that covered multiple dimensions of risk factors and established a novel and comprehensive nomogram model. Decision curve analysis (DCA) and a clinical impact curve (CIC) were used to validate the clinical usefulness and applicability net benefits of the model.

## Materials and methods

### Patients

**Fig. 1:**
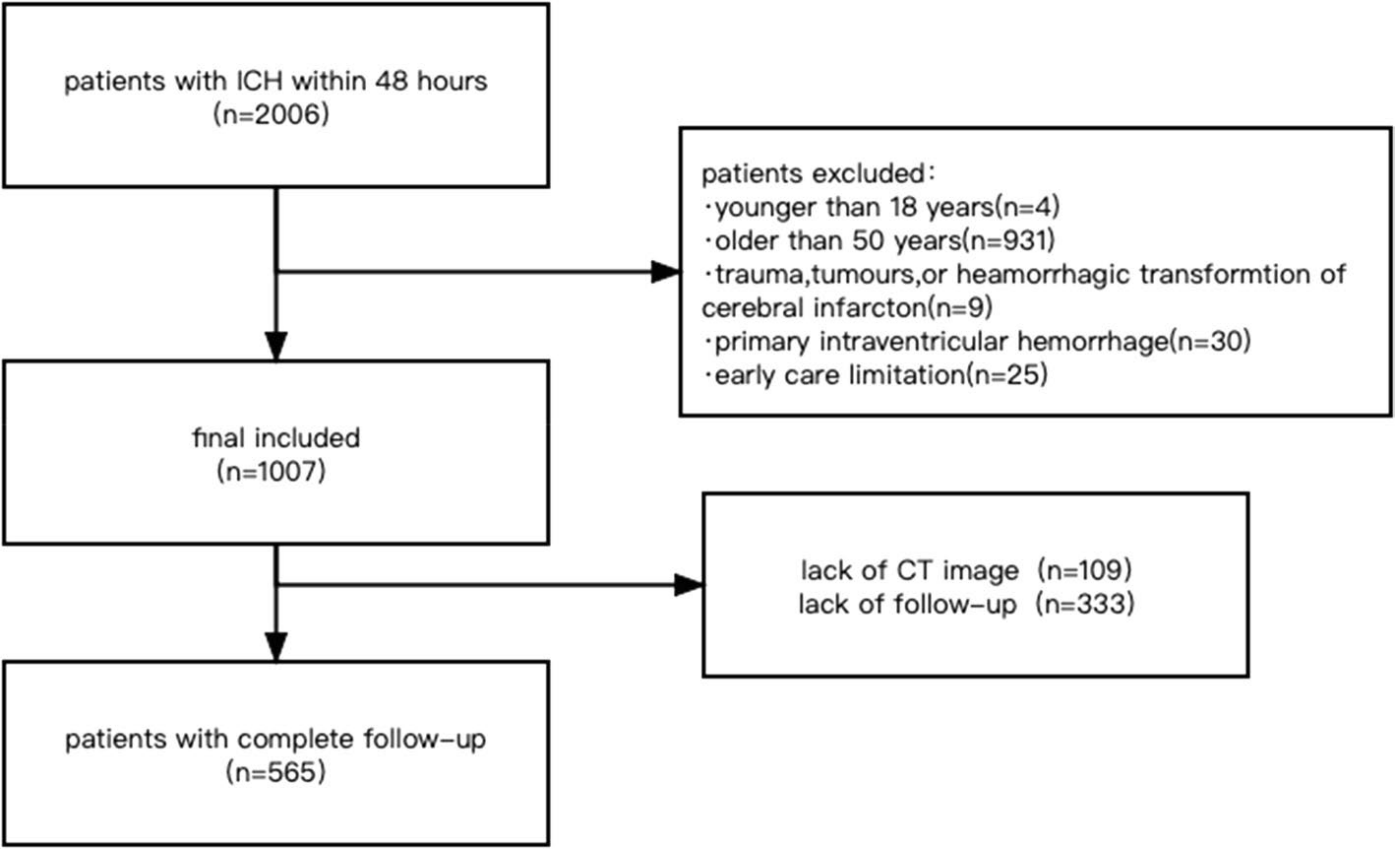
Flow chart for retrospective enrolment of patients

This study retrospectively analysed nontraumatic first-ever ICH patients between 18 and 50 years of age treated at Tongji Hospital affiliated with Tongji Medical College of Huazhong University of Science and Technology from January 1, 2012, to December 31, 2018. ICH was diagnosed according to the WHO criteria and confirmed by brain noncontrast CT [14]. We excluded ICH patients caused by trauma, tumours, primary subdural/epidural/subarachnoid hemorrhage, and postinfarct haemorrhagic transformation. Our study was approved by the institutional ethics Committee of Tongji Medical College of Huazhong University of Science and Technology. The enrolment flow chart is shown in Fig. 1.

### Data collection

Baseline data, including age, sex, history of diseases, smoking and drinking status, hematoma features, laboratory test data, systolic and diastolic blood pressure (BP), treatment and clinical assessment scales on admission, were collected. hematoma features consisted of hematoma volume (calculated for the ABC/2 method [15]), hematoma location, intraventricular extension and subarachnoid space extension. Laboratory test data included WBC counts, haemoglobin level, PLT counts, liver function, kidney function and blood glucose on admission. Treatment means conservative treatment and surgical treatment, including ventricular drainage, craniotomy or minimally invasive hematoma evacuation.

On admission, four clinical assessment scales were used: the National Institutes of Health Stroke Scale (NIHSS), ICH score, GCS (Glasgow Coma Scale) and ICH-FOS score(ICH Functional Outcome Score) [16–18].

Patients were followed through the death date or the last follow-up date (August 31, 2019) by telephone interview. We used the modified Rankin Scale (mRS) to evaluate the patient's outcome at 6 months. Favourable and unfavourable functional outcomes were defined as mRS ≤ 3 and mRS > 3, respectively.

### Statistical analysis

Continuous variables were reported as the means ± SD or medians (IQR). Categorical variables were reported as n (%). For univariate analysis, differences between the two groups were assessed by the Mann‒Whitney U test or Student’s t test for continuous variables and Fisher’s exact test or the χ^2^ test for categorical variables. All variables with a probability value < 0.1 in the univariate analysis were entered into a multivariate logistic regression analysis.

The nomogram model was established on those predictors in multivariate analysis with the package “rms” in R. To verify the performance of the nomogram model, the area under the curve (AUC) of the receiver-operating characteristic (ROC) and Harrell’s concordance index (C-index) were calculated. Calibration was carried out using a calibration plot, in which the predicted probabilities were plotted against the frequency of the observed unfavourable outcome. The prediction of a well-calibrated model should be mirrored by a 45° diagonal line.

To compare different prognostic models, decision curve analysis (DCA) probabilities and clinical impact curves (CICs) were conducted to quantify the net benefits of different thresholds to evaluate the clinical value of the nomogram. The CIC was developed using the bootstrap resampling method (times = 1,000).

The statistical analysis was carried out using SPSS version 26.0 (IBM Corporation, Armonk, NY, USA) and the statistical software package R, version 3.5.2 (R Development Core Team, Auckland, New Zealand). A *P* value < 0.05 indicated a statistically significant difference.

## Results

### Patient characteristics

In this cohort, 565 patients were retrospectively recruited (male: 68.1%, female:31.9%; mean age: 42.6 ± 7.1 years; Fig. 1). The baseline data clinical data, history of diseases, and laboratory data of the patients was obtained from medical records (Table 1). We compared the baseline characteristics and mRS scores at 6 months, and favourable and unfavourable functional outcomes were defined as mRS ≤ 3 and mRS > 3. At the significance level of *p* = 0.05, age, sex, hematoma volume, history of hypertension, blood glucose, WBC counts, haemoglobin, AST and surgical treatment were related to unfavourable outcomes (Table 1). For the prognostic scores, NIHSS, GCS, ICH score and ICH-FOS score divided cases into different functional outcome groups with high statistical significance.

**Table 1:** Participant characteristics and univariate analysis to identify independent predictors of functional outcome in young adults 6-months post-intracerebral haemorrhage. (Continuous variables were reported as the means ± SD or medians (IQR). Categorical variables were reported as n (%))

	All patients (*n* = 565)	Favourable outcome (*n* = 448)	Unfavourable outcome (*n* = 117)	*P* value
Demographics				
Age (ys)	42.6 ± 7.1	42.2 ± 7.3	44.2 ± 6.5	< 0.001^***^
Sex				< 0.01^**^
Male sex	385(68.1%)	317(70.8%)	68(58.1%)	
Female sex	180(31.9%)	131(29.2%)	49(41.9%)	
Vascular cause	70(12.4%)	58(12.9%)	12(10.3%)	0.427
History of diseases				
Hypertension	331(58.6%)	253(56.5%)	78(66.7%)	< 0.05^*^
Diabetes mellitus	29(5.1%)	23(5.1%)	6(5.1%)	0.998
Coronary heart disease	11(2.0%)	9(2.0%)	2(1.7%)	1
Atrial fibrillation	2(0.4%)	25(5.6%)	3(2.6%)	0.306
Oral anticoagulation	8(1.4%)	5(1.1%)	3(2.6%)	0.459
Previous stroke	45(8.0%)	35(7.8%)	10(8.5%)	0.795
Lifestyle				
Heavy smoking	178(31.5%)	143(31.9%)	35(29.9%)	0.678
Alcohol abuse	157(69.6%)	130(29%)	27(23.1%)	0.201
Hematoma volume(ml)	13.7(5.8–29.6)	12.1(4.9–26.3)	21.6(8.4–41.4)	< 0.001^***^
Hematoma location				
Lobar lesion	164(29.0%)	127(28.3%)	37(31.6%)	0.487
Basal ganglion lesion	145(25.66%)	113(25.2%)	32(27.4%)	0.639
Infratentorial lesion	38(6.73%)	26(5.8%)	12(10.3%)	0.087
Multiple hemorrhages	6(1.0%)	4(0.9%)	2(1.7%)	0.443
Intraventricular extension	146(25.8%)	109(24.3%)	37(31.6%)	0.304
Subarachnoid space extension	45(8.0%)	33(7.4%)	12(10.3%)	0.109
Systolic blood pressure (mmHg)	152(135–171)	152(133–171)	152(137–177)	0.606
Diastolic blood pressure (mmHg)	94(81–107)	94(81–107)	94(82–108)	0.926
Laboratory data at admission				
Blood glucose(mg/dl)	111.6(95.4–135)	108(93.6–120.6)	120.6(104.4–167.4)	< 0.001^***^
WBC(10^9^/L)	10.0(7.4–12.9)	9.7(7.3–12.3)	11.9(8.5–15.1)	< 0.001^***^
Hemoglobin(mg/dl)	14.4(13.2–15.4)	14.4(13.3–15.4)	14.3(12.6–15.1)	< 0.01^**^
PLT(10^9^/L)	207(175–245)	205(175–245)	207(162–249)	0.997
AST(U/L)	19(15–26)	19(15–24`)	22(16–31)	< 0.01^**^
eGFR, mL/min/1.73m^2^	102.4(82.2–114.2)	102.7(83.5–115.7)	97.7(67.2–112.3)	0.105
Surgical treatment	172(30.44%)	123(27.5%)	49(41.9%)	< 0.01^**^
GCS	14(10–15)	14(12–15)	10(7–14)	< 0.001^***^
NIHSS	11(4–16)	9(3–15)	18(11–25)	< 0.001^***^
ICH score	1(1–2)	1(1–2)	2(1–3)	< 0.001^***^
ICH-FOS	3(1–5)	3(1–5)	6(3–8)	< 0.001^***^

*Abbreviations*: *AST* Aspartate aminotransferase, *NIHSS* National Institutes of Health Stroke Scale, *GCS* Glasgow Coma Scale, *ICH-FOS* ICH Functional Outcome Score

### Development and validation of the nomogram

In multivariate logistic regression analysis (Table 2), six factors were found infratentorial significantly associated with unfavourable outcomes,lesions (adjusted odds ratio [aOR] = 3.708, 95% confidence interval [CI], 1.490–9.227; *P* = 0.005) were most significantly associated with unfavourable outcomes.These variables were independent of each other.

**Table 2:** Multivariable logistic regression analysis to identify independent predictors of functional outcome in young adults 6-months post-intracerebral haemorrhage

	OR	95%CI	*P*-value
Age	1.054	1.014–1.096	0.01^**^
Hematoma volume	1.014	1.002–1.027	< 0.05^*^
Blood glucose	1.135	1.037–1.241	0.01^**^
Infratentorial lesion	3.708	1.490–9.227	0.01^**^
Hemoglobin	0.981	0.969–0.993	0.01^**^
NIHSS	1.105	1.069–1.141	< 0.001^***^

Multivariable logistic regression adjusted for age, male sex, history of hypertension, hematoma volume, infratentorial lesion, WBC count, blood glucos, PLT, AST, eGFR,systolic blood pressure, suigical treatment, NIHSS and GCS

*Abbreviations*: *NIHS*S National Institutes of Health Stroke Scale; *OR* Odds ratio, *CI* Confidence interval

**Fig. 2:**
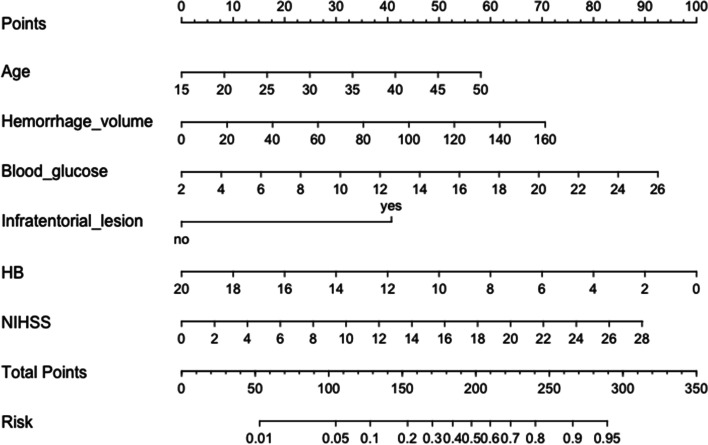
Nomogram of the study population to predict poor functional outcome in young adults 6-months post-intracerebral haemorrhage. Abbreviations: NIHSS, National Institutes of Health Stroke Scale; HB, hemoglobin level

**Fig. 3:**
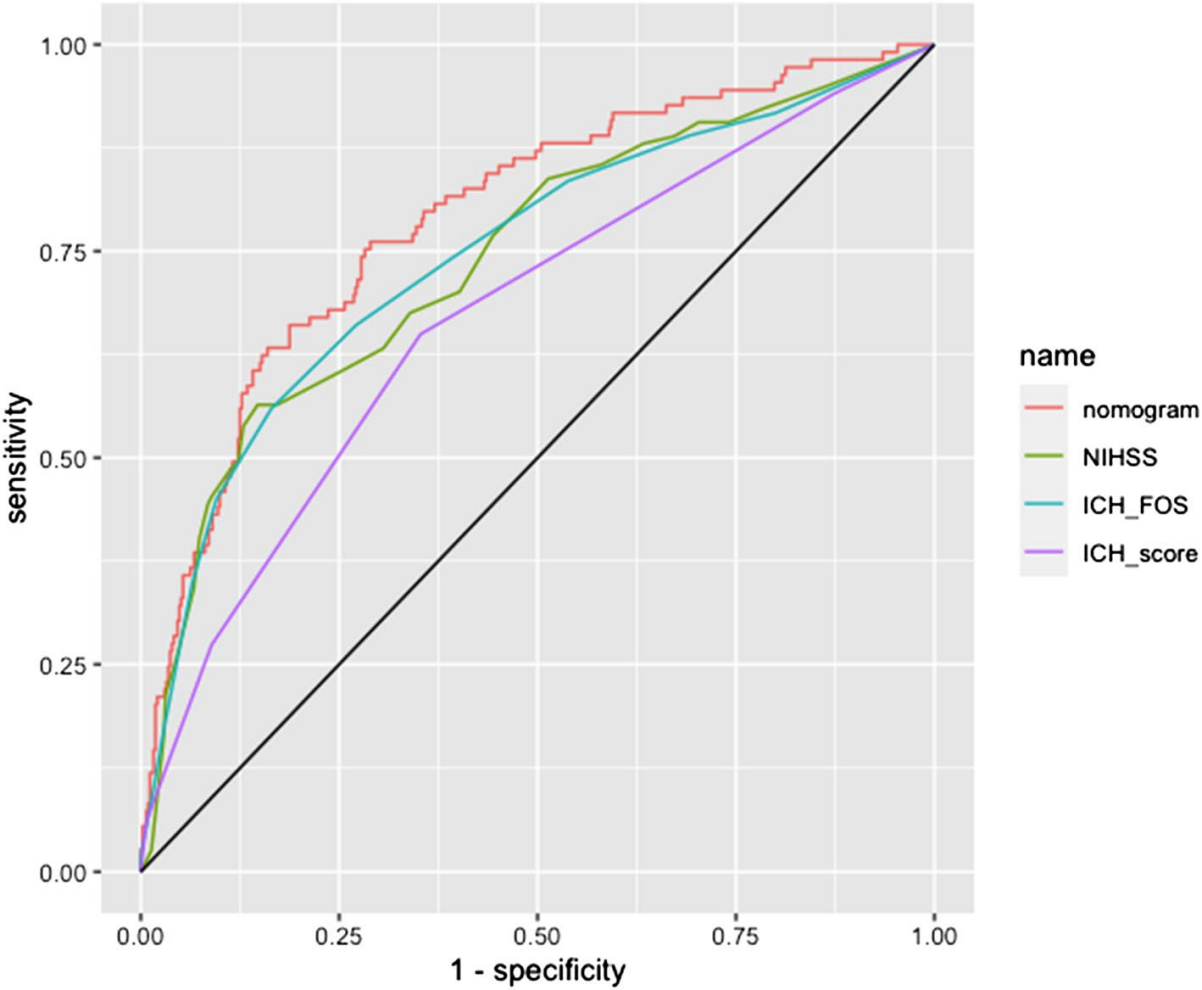
Area under the receiver operating characteristic curve (AUROC) displaying the predictive accuracy (sensitivity, specificity) of the nomogram compared with NIHSS, ICH-FOS and ICH score.NIHSS = National Institutes of Health Stroke Scale score

**Fig. 4:**
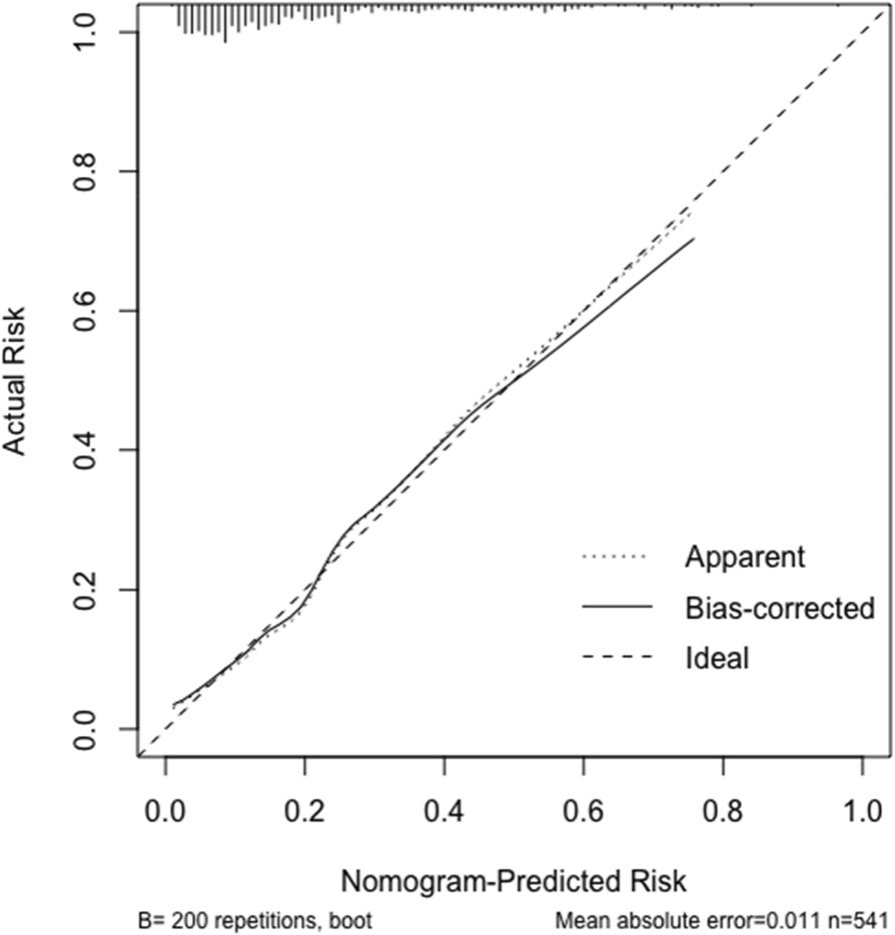
Calibration curve for the nomogram.The dashed line represents the reference line where an ideal nomogram would lie. The dotted line is the performance of nomogram. The solid line corrects for any bias in the nomogram

Based on the above six independent risk factors, we established a nomogram to estimate the prognosis of intracerebral hemorrhage in younger adults at 6 months (Fig. 2). The nomogram was developed by assigning a graphic initial score to each of the 6 independent prognostic factors with a point range from 0 to 100, which was then summed to create a total score and finally converted into an individual risk of 6-month unfavourable outcome expressed as a percentage, thus ranging from 0 to 100%. It was predicted that a higher total score of the nomogram was associated with a higher likelihood of unfavourable outcomes, while a lower total score was associated with a lower likelihood of unfavourable outcomes. Validation of the nomogram was accomplished by 200 bootstraps. For the logistic binary variable model, the C-index is equivalent to the area under the ROC curve. The C-index of the nomogram was 0.791 (95% CI, 0.743–0.840) higher than the AUCs of the NIHSS score (0.742, 95% CI, 0.688–0.795), ICH-FOS (0.764, 95% CI, 0.691–0.801) and ICH score (0.672, 95% CI, 0.619–0.726) (Fig. 3). Furthermore, the calibration curve revealed full fit of the nomogram to predict the actual risk of an unfavourable outcome, indicating that the prediction results were accurate (Fig. 4).

### Decision curve analysis and clinical impact curve for the nomogram

**Fig. 5:**
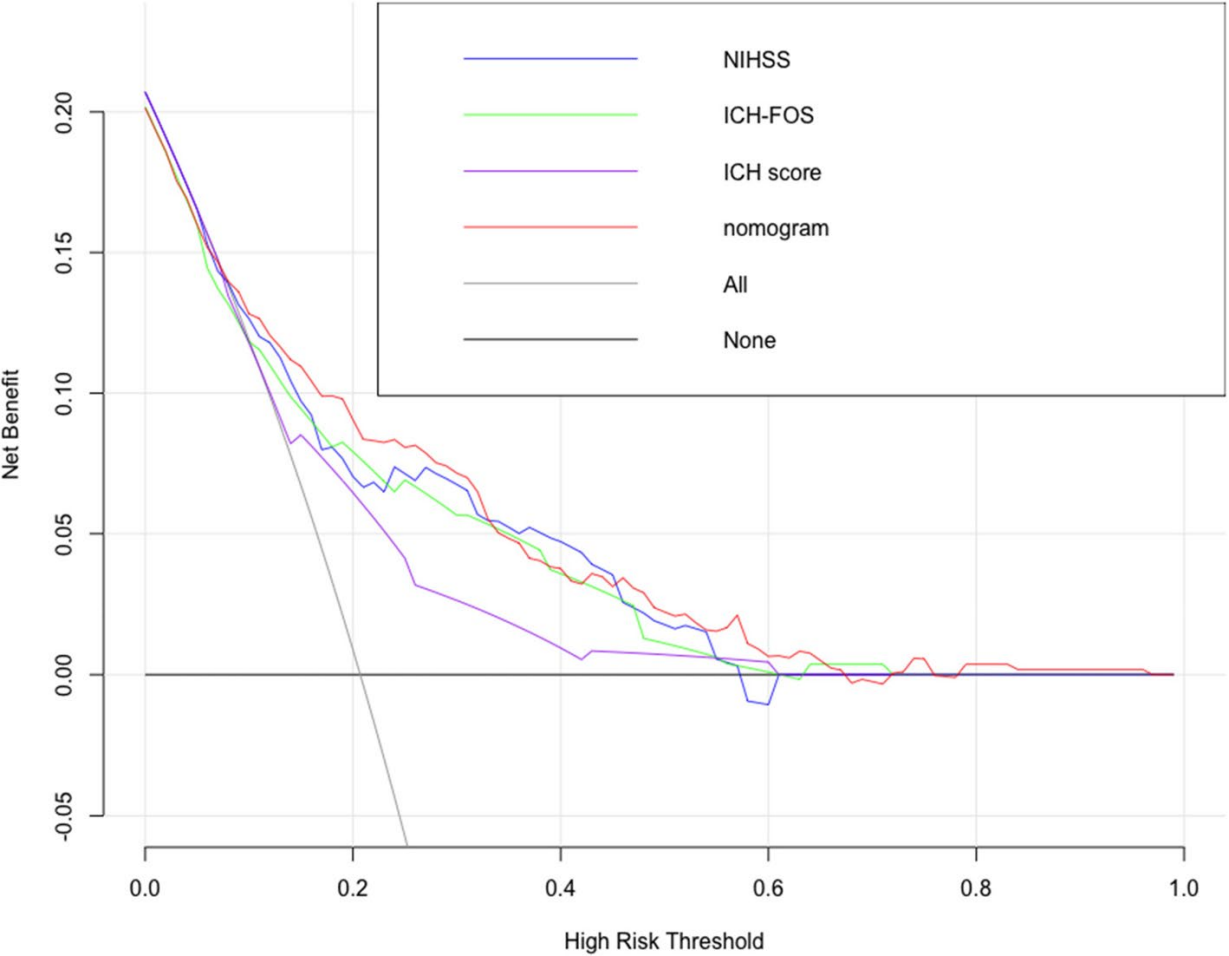
Decision curve analysis of the nomogram compared with NIHSS, ICH-FOS and ICH score. The x-axis indicates the threshold for critical care outcome. The y-axis indicates the net benefit of clinical decision. NIHSS = National Institutes of Health Stroke Scale score

**Fig. 6:**
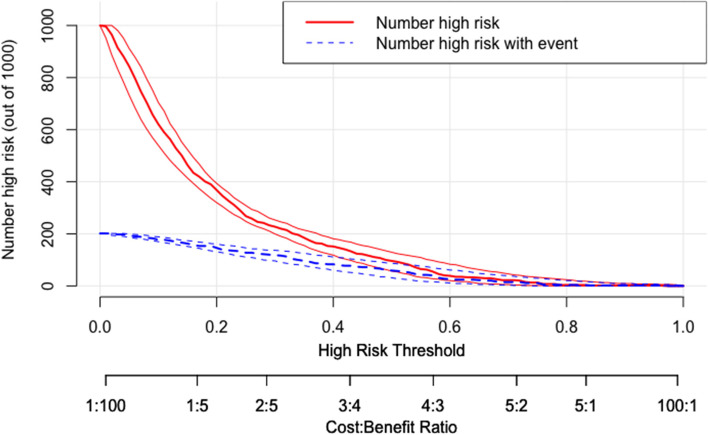
Clinical impact curve (CIC) of nomogram model. The red curve (number of high-risk individuals) indicates the number of people who are classified as positive (high risk) by the model at each threshold probability; the blue curve (number of high-risk individuals with outcome) is the number of true positives at each threshold probability. CIC visually indicated that the nomogram conferred high clinical net benefit and confirmed the clinical value of the nomogram model

In the last step, as shown in Fig. 5, when the high-risk threshold was between 0.1–0.7, the nomogram model could obtain a net benefit. Meanwhile, between 0.1–0.3, its performance was better than all other 3 models. Based on the DCA, a CIC using the cost:benefit ratio to evaluate the nomogram can quickly help us understand the significance of the nomogram in predicting the 6-month prognosis. We then estimated the number of patients with unfavourable prognosis for each risk threshold and established the proportion of those who are true positives (Fig. 6).The red curve (number of high-risk individuals) indicates the number of people who are classified as positive (high risk) by the model at each threshold probability; the blue curve (number of high-risk individuals with outcome) is the number of true positives at each threshold probability. CIC visually indicated that the nomogram conferred high clinical net benefit and confirmed the clinical value of the nomogram model.For example, if a 20% risk threshold was used, then of 1000 people screened, approximately 400 would be deemed at high risk of unfavourable prognosis,with approximately 180 of these being true cases.

## Discussion

To our knowledge, the present study is to establish a nomogram model to predict 6-month prognosis in younger adults with ICH. In this cohort, age, hematoma volume, blood glucose, infratentorial lesion, haemoglobin and the NIHSS score were significant prognostic factors in the univariate logistic regression analysis and were confirmed as independent risk factors for functional prognosis. Based on those predicting parameters, we constructed a nomogram model for evaluation. We incorporated several common clinical factors and an easily overlooked blood indicator haemoglobin into our model. The performance of the present nomogram was strictly assessed and internally validated, and its net benefit was also explored by DCA and the CIC compared with other prognostic scores commonly used in clinical practice. In addition, our study demonstrates that the clinical applicability of this nomogram is feasible for ICH in younger adults.Estimating haemoglobin levels is part of standard blood analysis. Establishing a link between haemoglobin levels and the undesirable prognosis of stroke at no extra cost or the need for additional tests suggests this could be a widely-implemented clinical screening tool.

Haemoglobin level is part of the automated analysis of blood cells at no additional cost, establishing an important link with the undesirable prognosis of stroke [19]. In recent years, several studies have found a positive correlation between anaemia and higher mortality in stroke patients [12, 20]. In 2018, a meta-analysis identified seven cohort studies with 7,328 ICH patients, including 1,546 patients with anaemia, revealing that anaemia was associated with an increased risk of poor outcome in patients with ICH (OR = 2.29 for 3-month outcome, 95% CI 1.16 to 4.51; OR = 3.42 for 12-month outcome, 95% CI 0.50 to 23.23) [12]. Another recent study that reported a large meta-analysis of pooled data from the ATACH-2, FAST, and ERICH studies also found that higher admission Hb levels were associated with better outcomes [20]. It has been posited that such a result may have occurred because these patients had a haematologic disorder that causes the hematoma and, eventually, a poor prognosis [21]. Lower erythrocyte counts may result in less efficient radial transport of platelets towards the vessel wall, preventing the platelet endothelial interaction that is vital to haemostasis initiation. In addition, erythrocytes themselves may be implicated in haemostasis through their adhesion to the injured vessel wall in addition to their interaction with platelets and fibrinogen, leading to blood clot contraction [22]. David J. Roh et al. also suggested that hyperacute transfusion of pRBCs can be considered in preventing the early occurrence of HE to improve outcomes. However, the timing of red blood cell transfusions still needs more research to be clarified [21].

Previous studies have suggested that hyperglycaemia is associated with mortality in ICH patients [23, 24]. A meta-analysis of 16 studies reinforced this view: high blood glucose was significantly associated with poor functional outcome in ICH patients [25]. Previous animal studies identified an evident association between hyperglycaemia and perihematomal neuronal apoptosis in rat models [26]. In ICH models, hematoma with high blood glucose was found to lead to neurological injury and decreased autophagy [27]. High blood glucose can increase superoxide production in ICH induced by tissue plasminogen activator [28].

A 2013 study showed that age could affect the prognosis of intracerebral hemorrhage in younger people, and the INTERACT-2 study also showed that age is a strong predictor of a poor prognosis for intracerebral hemorrhage, consistent with the results of this study [11, 10]. The reason for this may be that younger people are in better physical condition than elderly people. Their vascular atherosclerosis is mild, and they can establish collateral circulation in a short period of time so that angioedema is relatively mild, neurological deficits are milder, and younger patients have a strong sense of health care and actively carry out secondary prevention [29].

The GCS and NIHSS scores are commonly used stroke scales, with GCS scores assessing a patient's state of consciousness and NIHSS scores assessing both the patient’s state of consciousness and neurological deficits. A 2003 study found that the NIHSS score was superior to the GCS score in predicting the prognosis of patients with intracerebral hemorrhage. This is consistent with the results of the univariate analysis of this study that the NIHSS score and GCS score affected the prognosis at the time of univariate analysis, while the NIHSS score was independent of the influencing factors in the multivariate regression analysis [30].

Using such 6 variables, a nomogram combining haemoglobin with acceptable discrimination (C-index = 0.791) and calibration was established for predicting an unfavourable outcome, and it seems to possess more power efficiency than currently utilized prognostic tools. The decision curve suggested that, when the probability ranged from 20 to 40%, the net benefits of the nomogram were higher than those of the ICH-score and ICH-FOS. Moreover, the outcome was verified by a clinical impact curve.

In this study, our nomogram is novel and shows certain advantages. First, we integrated and internally validated a new nomogram model that combines clinical scores and laboratory data. The nomogram can be employed to predict early functional prognosis with high accuracy (AUC 0.791). Second, DCA and CIC were used to creatively evaluate the clinical performance of the new model. Finally, haemoglobin levels at admission may be an easily overlooked prognostic risk factor for negative outcomes after ICH. Our novel nomogram model may provide a promising and convenient tool to predict the early functional prognosis in younger adults with ICH. Prospective, multicentre studies are needed to validate these findings.

However, this study has limitations. First, it was a retrospective study in a single centre and not a randomized controlled trial (RCT). As a result, selection bias caused by single-centre data may have resulted in lack of broad representation of results. The accuracy of clinical valuation may have been attenuated by its retrospective nature. External validations in other institutions are warranted. Moreover, our model covered many types of clinical data variables, but the lack of detailed neuroimaging and therapeutic data may have led to an unavoidable systemic bias that weakens the discriminative performance of the nomogram. Finally, we collected a limited number of cases and had a 30% loss-of-follow-up rate, which may have affected the credibility of the results. Despite these limitations, we made a first attempt to establish and validate a nomogram model to predict a 6-month functional prognosis in younger ICH patients.

## Conclusion

In summary, the study shows that age, hematoma volume, blood glucose, infratentorial lesions, haemoglobin and the NIHSS score are associated with unfavourable outcomes in younger ICH patients. The haemoglobin level at admission may be an easily overlooked factor.

The nomogram constructed from these data could be a promising and convenient tool to predict the early functional prognosis of younger people with ICH. In addition, more prospective multicentre studies are needed to confirm these findings.

## Data Availability

The datasets presented in this article are not readily available because further data mining is ongoing. Requests to access the datasets should be directed to wangfurong.china@163.com.

## Abbreviations

ICH: Intracerebral hemorrhage
NIHSS: National Institutes of Health Stroke Scale score
GCS: Glasgow Coma Scale
ICH-FOS: CH Functional Outcome Score
DCA: Decision curve analysis
CIC: Clinical impact curve
mRS: Modified Rankin Scale
AUC: Area under curve
